# Supplementary material for: A powerful framework for differential co-expression analysis of general risk factors
Source: Bioinformatics. 2025 Oct 10;41(11):btaf565. doi: 10.1093/bioinformatics/btaf565 (PMC12619644; doi:10.1093/bioinformatics/btaf565)
Supplement: btaf565_Supplementary_Data [file btaf565_supplementary_data.pdf]

Supplementary materials: A powerful framework for differential co-expression analysis of general risk factors

Andrew J. Bass[1,*], David J. Cutler[2] and Michael P. Epstein[2,*]

[1] *Department of Medicine, University of Cambridge, Cambridge, CB2 0QQ, UK*
[2] *Department of Human Genetics, Emory University, Atlanta, GA 30322, USA*
[*] *Corresponding authors:* ab3105@cam.ac.uk, mpepste@emory.edu

Contents

1 Supplementary methods **2**
1.1 A general model for differential co-expression analysis 2

2 Supplementary figures **5**

3 Supplementary tables **14**

1 Supplementary methods

1.1 A general model for differential co-expression analysis

Differential co-expression in a pathway is a statistical phenomenon that arises from unmodeled biological variation involving an observed risk factor of interest and some unobserved (or latent) factor. Consequently, this unmodeled variation induces a correlation pattern between pairs of genes in a pathway that varies as a function of the risk factor. We first motivate the observation that such correlation patterns are due to a latent factor interacting with a risk factor, and then extend our discussion to a more general model.

Latent factors can induce differential correlation patterns between two genes. To simplify our discussion, consider the expression of two genes in a pathway denoted by Y_{j1} and Y_{j2} for $j = 1, 2, \dots n$ individuals. Let the risk factor of interest be denoted by X_j and the shared latent factor be denoted by W_j. The risk factor can be a continuous (e.g., age) or categorical (e.g., SNP or biological condition) variable, while the shared latent factor is some unobserved environmental factor that is difficult to measure. We assume that the risk and latent factors contribute to expression additively as

$$\begin{aligned} Y_{j1} &= \beta_1 X_j + \omega_1 W_j + \epsilon_{j1}, \\ Y_{j2} &= \beta_2 X_j + \omega_2 W_j + \epsilon_{j2}, \end{aligned} \tag{S1}$$

where β_1 and β_2 are the effect sizes of the risk factor, ω_1 and ω_2 are the effect sizes for the latent factor, and ϵ_{j1} and ϵ_{j2} are independent normally distributed random errors for gene 1 and 2, respectively.

If the latent factor interacts with the risk factor, and affects the expression of both genes, then a correlation pattern between the genes is induced. More specifically, assuming that the random errors are independent of the risk and latent factors, the individual-specific gene correlation (IGC) is

$$\mathrm{Cor}[Y_{j1}, Y_{j2} \mid X_j] = \tau_j \times \mathrm{E}\big[W_j^2 \mid X_j\big], \tag{S2}$$

where $\tau_j = \frac{\omega_1 \omega_2}{\sigma_{Y_{j1}|X_j} \sigma_{Y_{j2}|X_j}}$ is the effect size and $\sigma_{Y_{j1}|X_j}$ and $\sigma_{Y_{j2}|X_j}$ are the conditional standard deviations of gene 1 and 2, respectively. As an example, suppose the latent factor $W_j = X_j U_j$

is an interaction between the risk factor and an environmental factor, U_j, that follows a standard normal distribution. In this case, when the two genes share the latent factor (i.e., $\omega_1 \neq 0$ and $\omega_2 \neq 0$), the conditional correlation pattern $\mathrm{Cor}[Y_{j1}, Y_{j2} \mid X_j] = \tau_j X_j^2$ is induced and the pathway is classified as "differentially co-expressed." Note that the conditional variance is $\sigma^2_{Y_{j1}|X_j} = \sigma^2_{\epsilon_{j1}} + \omega_1^2 X_j^2$ and $\sigma^2_{Y_{j2}|X_j} = \sigma^2_{\epsilon_{j2}} + \omega_2^2 X_j^2$ for gene 1 and 2, respectively. Thus, our strategy to assess differential co-expression is to test whether the IGCs varies as a function of the risk factor in a pathway.

Inferring differential correlation patterns in a pathway. We extend the previous model to include multiple genes in a pathway. Consider a pathway with expression value Y_{jk} for $j = 1, 2, \ldots n$ individuals and $k = 1, 2, \ldots, r$ genes. Let $\boldsymbol{C}$ be a $n \times l_C$ matrix of covariates and $\boldsymbol{M}$ be a $n \times l_M$ matrix of batch effects. The gene expression model is

$$Y_{jk} = \beta_k X_j + \boldsymbol{C}_{j\cdot}\boldsymbol{\Gamma}_{\cdot k} + \boldsymbol{M}_{j\cdot}\boldsymbol{\Phi}_{\cdot k} + \omega_k W_j + \epsilon_{jk}, \tag{S3}$$

where $\boldsymbol{\Gamma}_{\cdot k}$ and $\boldsymbol{\Phi}_{\cdot k}$ are effect sizes for $\boldsymbol{C}_{j\cdot}$ and $\boldsymbol{M}_{j\cdot}$, respectively, ϵ_{jk} is a normally distributed random variable whose variance can depend on the risk factor (i.e., heteroskedasticity), and the subscript '$\cdot$' is a placeholder to represent all of the columns (or rows) of a matrix. To illustrate our strategy, we assume the effect sizes are known. We can then calculate the residuals as

$$\begin{aligned} e_{jk} &= Y_{jk} - \beta_k X_j - \boldsymbol{C}_{j\cdot}\boldsymbol{\Gamma}_{\cdot k} - \boldsymbol{M}_{j\cdot}\boldsymbol{\Phi}_{\cdot k}, \\ &= \omega_k W_j + \epsilon_{jk}. \end{aligned} \tag{S4}$$

Since the risk factor may induce variance-specific effects, the residuals should be standardized to prevent false discoveries for differential co-expression testing, i.e., $\tilde{e}_{jk} = e_{jk}/s_{jk}$ where $s_{jk} = \sigma_{Y_{jk}|X_j, \boldsymbol{C}_{j\cdot}, \boldsymbol{M}_{j\cdot}}$ is the conditional standard deviation. Note that variance effects from other terms should also be modeled when standardizing the residuals. Failing to model the variance-specific effects of the primary variable will lead to an incorrect empirical null distribution in the permutation algorithm (Figure S4). More generally, modeling heteroskedasticity is important for valid inferences in any differential co-expression method.

We show in the main text that a latent interaction can be detected based on calculating the individual-specific gene covariances (IGC). For the jth individual, the conditional $r \times r$

individual-specific pathway covariance matrix is

$$\boldsymbol{\Sigma}_j = \begin{bmatrix} 1 & \mathrm{E}[\tilde{e}_{j1}\tilde{e}_{j2} \mid X_j, \boldsymbol{C}_{j\cdot}, \boldsymbol{M}_{j\cdot}] & \cdots & \mathrm{E}[\tilde{e}_{j1}\tilde{e}_{jr} \mid X_j, \boldsymbol{C}_{j\cdot}, \boldsymbol{M}_{j\cdot}] \\ \mathrm{E}[\tilde{e}_{j2}\tilde{e}_{j1} \mid X_j, \boldsymbol{C}_{j\cdot}, \boldsymbol{M}_{j\cdot}] & 1 & \cdots & \mathrm{E}[\tilde{e}_{j2}\tilde{e}_{jr} \mid X_j, \boldsymbol{C}_{j\cdot}, \boldsymbol{M}_{j\cdot}] \\ \vdots & \vdots & \ddots & \vdots \\ \mathrm{E}[\tilde{e}_{jr}\tilde{e}_{j1} \mid X_j, \boldsymbol{C}_{j\cdot}, \boldsymbol{M}_{j\cdot}] & \mathrm{E}[\tilde{e}_{jr}\tilde{e}_{j2} \mid X_j, \boldsymbol{C}_{j\cdot}, \boldsymbol{M}_{j\cdot}] & \cdots & 1 \end{bmatrix},$$

where the IGC terms are the $q = \binom{r}{2}$ off-diagonal elements. Using the results from the previous section, the IGC between the kth and k'th genes is

$$\begin{aligned} \mathrm{Cor}\big[Y_{jk}, Y_{jk'} \mid X_j, \boldsymbol{C}_{j\cdot}, \boldsymbol{M}_{j\cdot}\big] &= \mathrm{E}\big[\tilde{e}_{jk}\tilde{e}_{jk'} \bigm| X_j, \boldsymbol{C}_{j\cdot}, \boldsymbol{M}_{j\cdot}\big], \\ &= \tau_{jkk'} \times \mathrm{E}\big[W_j^2 \mid X_j, \boldsymbol{C}_{j\cdot}, \boldsymbol{M}_{j\cdot}\big], \end{aligned} \tag{S5}$$

where $\tau_{jkk'} = \frac{\omega_k \omega_{k'}}{s_{jk} s_{jk'}}$ is the effect size and s_{jk} and $s_{jk'}$ are the conditional standard deviations of gene k and k', respectively. A pathway is differentially co-expressed if $\tau_{jkk'} \neq 0$ for at least one IGC term. Our results imply that the latent factor W_j can induce correlation patterns in a pathway whenever the factor is correlated with the risk factor and impacts the expression of the genes. Note that the above results can easily be extended to include multiple general risk factors and latent factors.

The KDCA framework estimates the IGC terms in a pathway by taking the cross products of the standardized residuals. We then use the cross products (see ref. [1] for theoretical details) to test for any evidence of differential co-expression in at least one of the IGC terms. One important topic when using KDCA involves the latent factor W_j. The latent factor(s) is a key component in any differential co-expression analysis as it captures unmeasured variability that can impact the correlation between genes. A challenge is that a latent factor(s) can represent biological and/or technical (i.e., batch) variability. Ideally, the technical factors are included in the model as an adjustment variable to avoid false discoveries. However, distinguishing latent technical and biological factors is a very difficult problem, and it is currently unclear how to appropriately account for technical factors (without removing biological variation) in differential co-expression analysis. Here, we inferred the technical factors using the approach in ref. [2] and included them as adjustment variables in our procedure. While this approach helps control for false discoveries due to technical factors, it may also remove some of the biological factors.

2 Supplementary figures

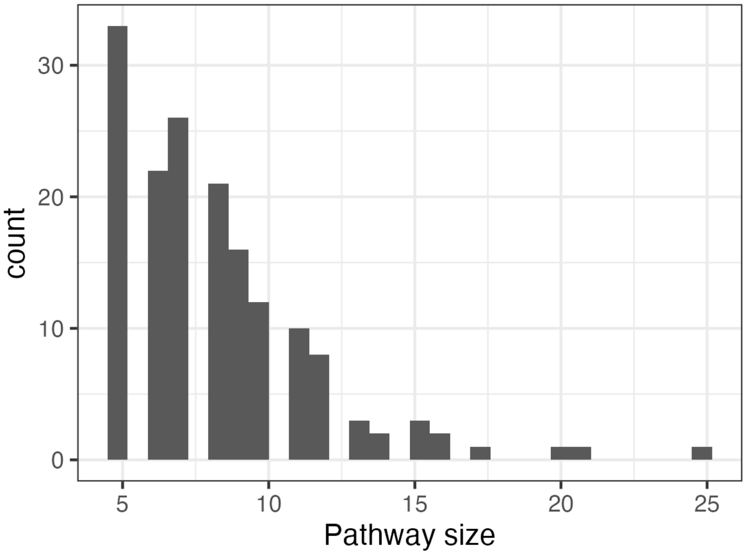

Figure S1: A histogram of the pathway sizes (i.e., number of genes) from the BioCarta database.

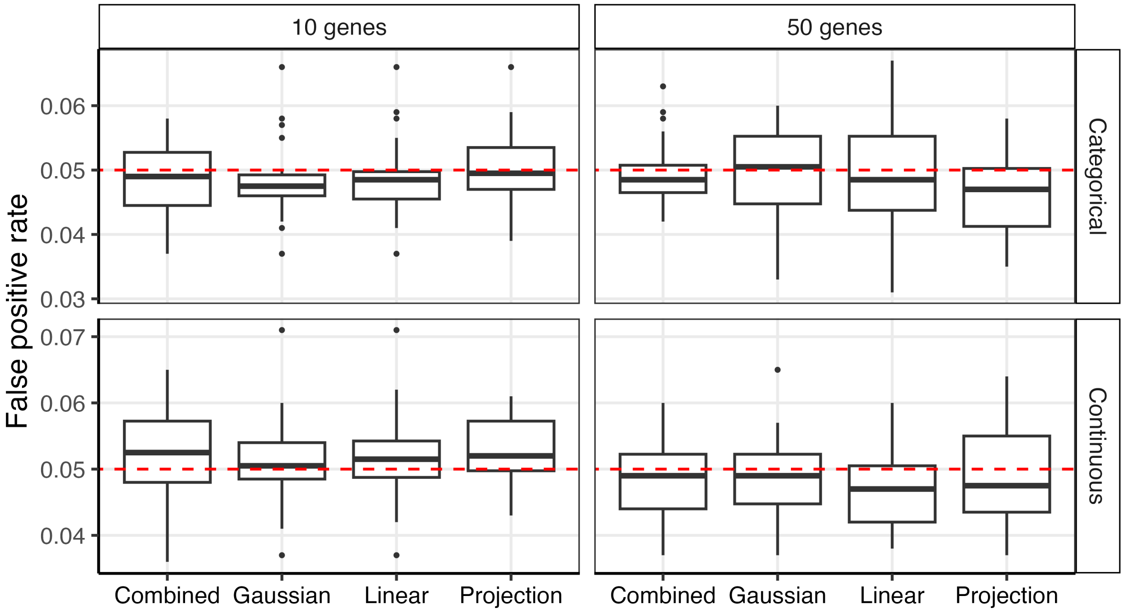

Figure S2: False positive rate of KDCA under the null hypothesis of no differential co-expression using the Gaussian, linear, and projection kernels when the risk factor is categorical (top row) or continuous (bottom row). We also considered an implementation that maximized power by combining all three kernels (combined). At each pathway size (columns), there were 20 simulated data sets with $1{,}000$ pathways and the empirical false positive rate was assessed at a type I error rate of 0.05 (red line).

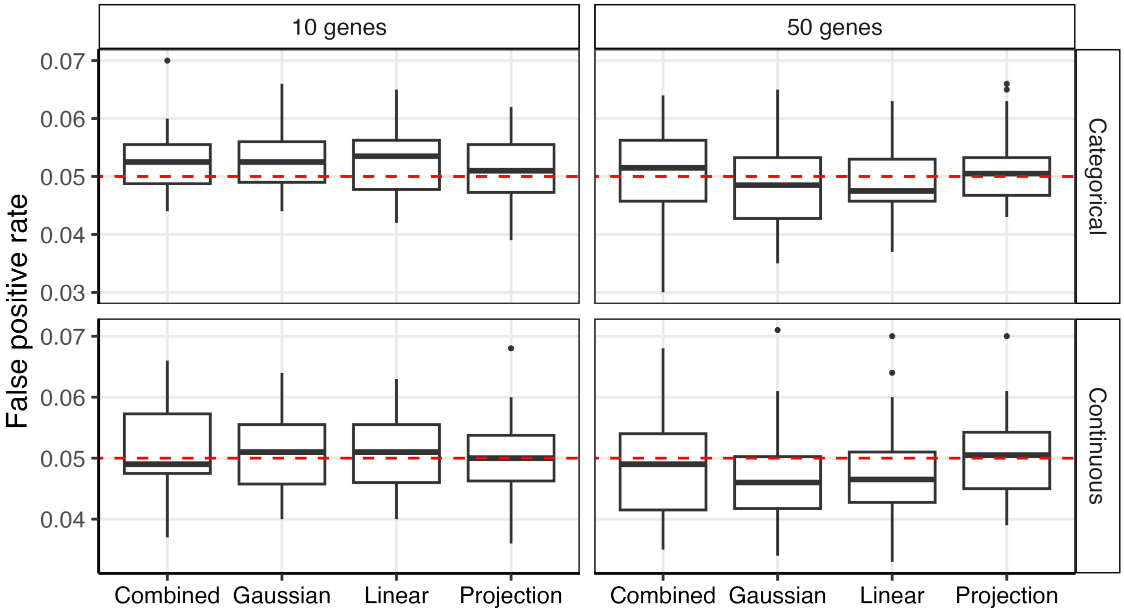

Figure S3: Assessing type I error rate in the RNA-seq simulation setting. We applied KDCA using the Gaussian, linear, and projection kernels to categorical (top row) and continuous (bottom row) risk factors. We also considered an implementation that maximized power by combining all three kernels (combined). At each pathway size (columns), there were 20 simulated data sets with $1{,}000$ pathways and the empirical false positive rate was assessed at a type I error rate of 0.05 (red line).

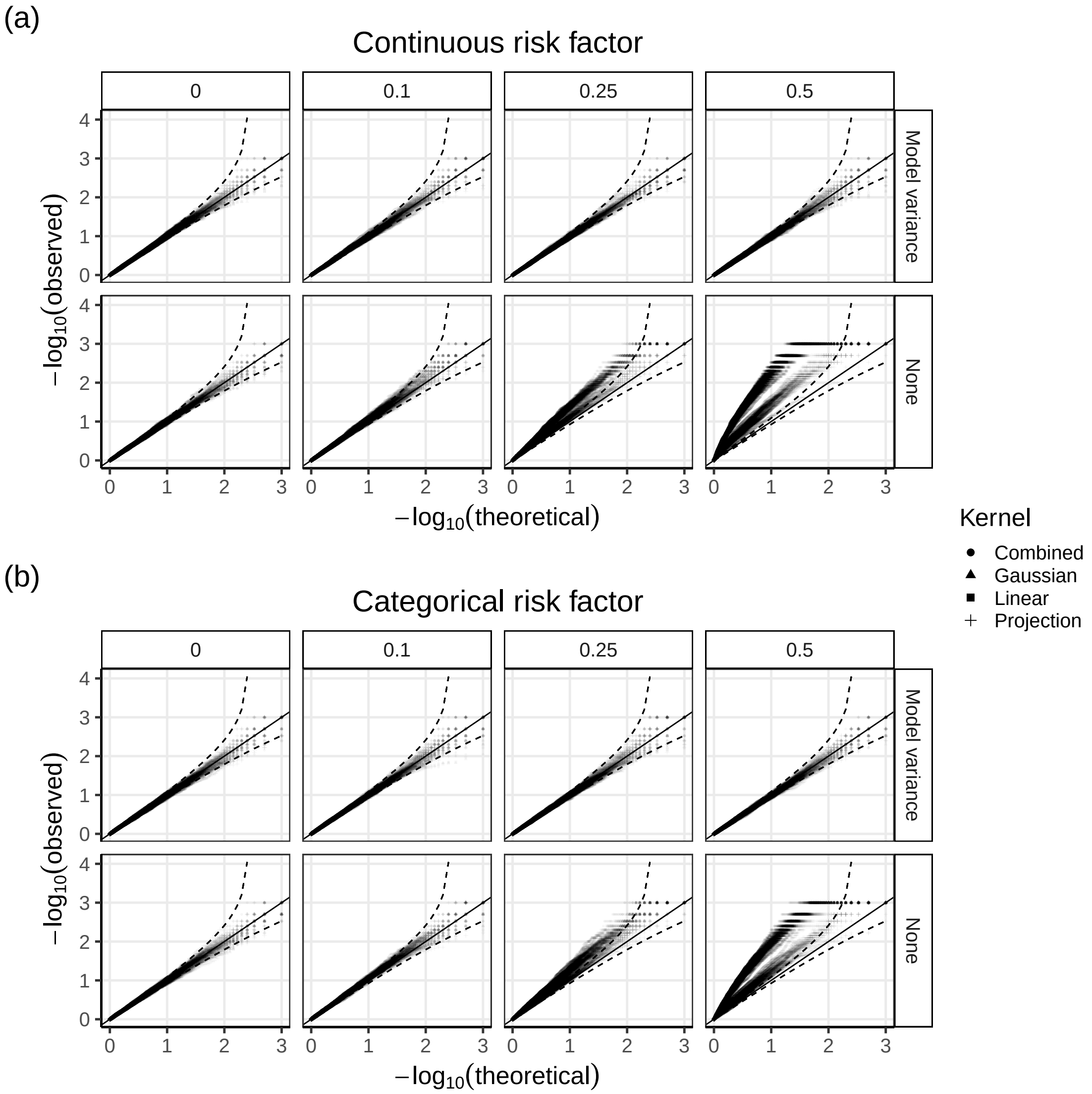

Figure S4: Evaluating variance adjustment in KDCA for continuous and categorical variables. The variance effect size was varied as $\mathrm{Uniform}(0, \delta')$ for $\delta' = 0, 0.1, 0.25,$ and 0.5. We simulated a (a) categorical and (b) continuous primary variable with 20 replicates per setting. We compared variance adjustment in KDCA ('Model variance') to no variance adjustment.

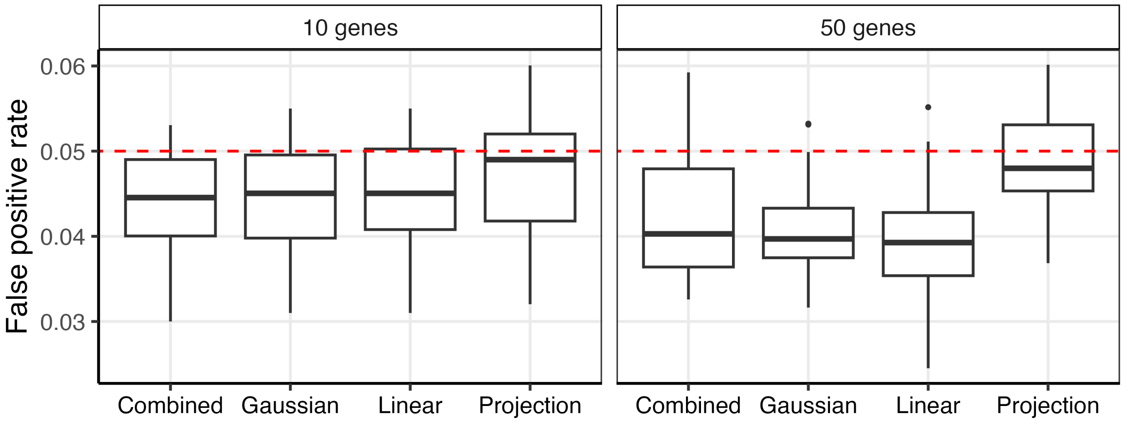

Figure S5: Assessing type I error rate when testing three risk factors for differential co-expression. We applied KDCA using the Gaussian, linear, and projection kernels to three primary variables. We also considered an implementation that maximized power by combining all three kernels (combined). At each pathway size (columns), there were 20 simulated data sets with $1{,}000$ pathways and the empirical false positive rate was assessed at a type I error rate of 0.05 (red line).

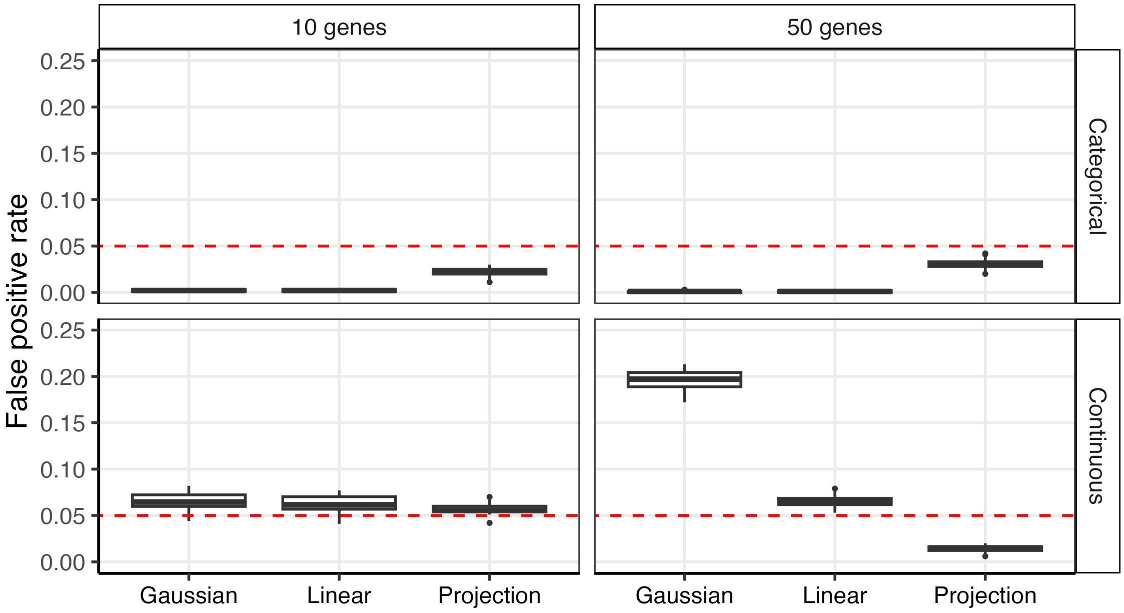

Figure S6: False positive rate of KDCA using a theoretical null approximation for the Gaussian, linear, and projection kernels when the risk factor is categorical (top row) or continuous (bottom row). At each pathway size (columns), there were 20 simulated data sets with $1{,}000$ pathways and the empirical false positive rate was assessed at a type I error rate of 0.05 (red line).

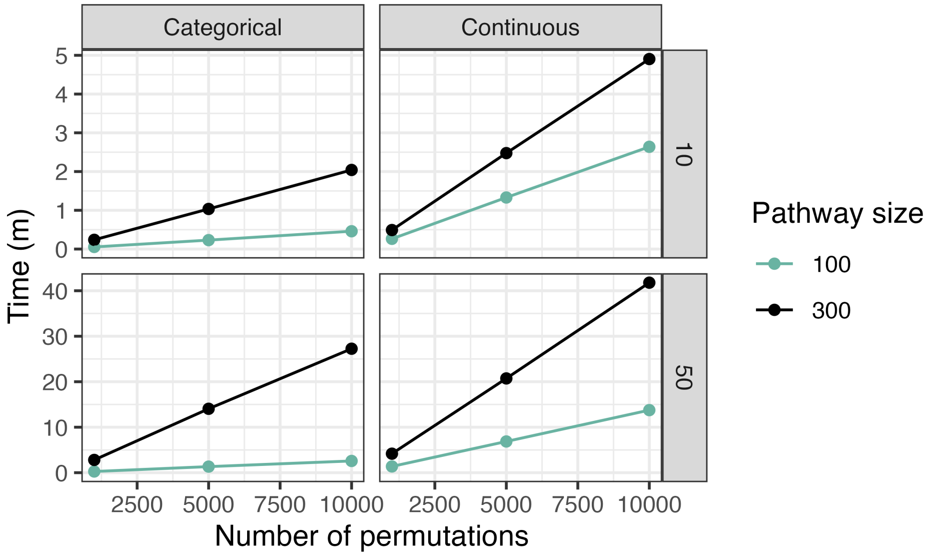

Figure S7: The computational time of KDCA with $1{,}000$ permutations as a function of the sample size (x-axis), pathway size (color), and risk factor type (columns) in the simulation study (normal setting). There were 50 replicates at each setting and the simulations were performed on a single core of an Apple M3 processor.

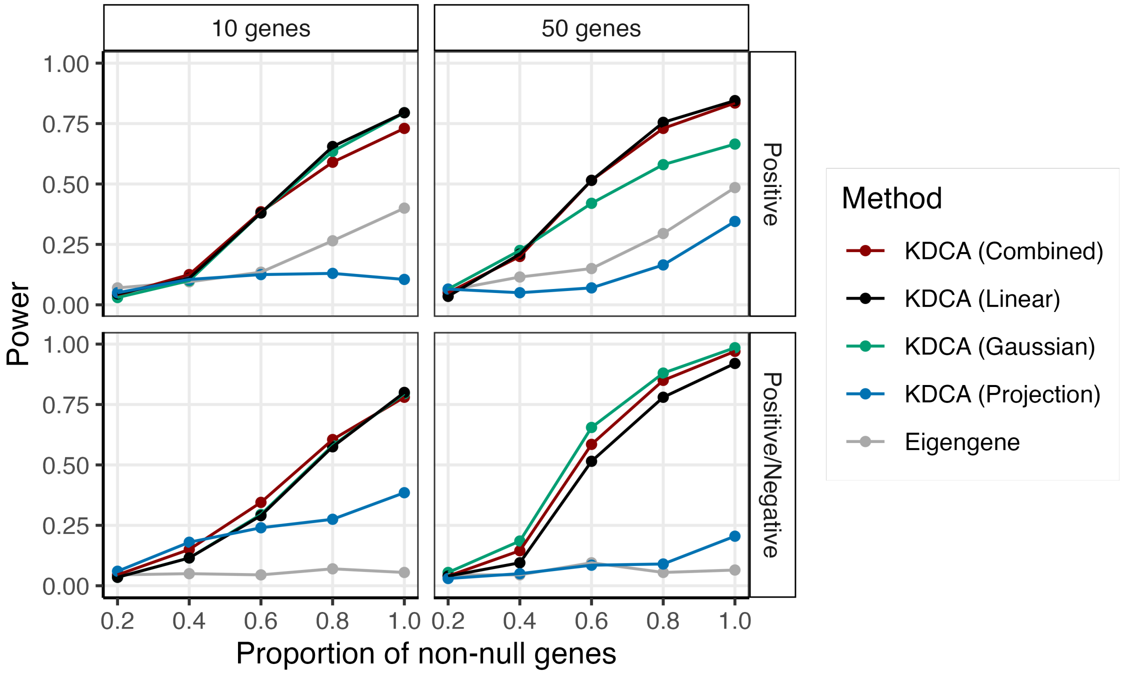

Figure S8: The empirical power of KDCA using the Gaussian (green), linear (black), and projection (blue) kernels when there are three risk factors. We also compared an implementation of KDCA that maximized power across all kernels (combined; red) to a standard eigengene (grey) approach. Our simulation study varied the pathway size (columns) and the type of differential co-expression (rows). Each point is the empirical power from 200 simulations at a significance threshold of 0.05.

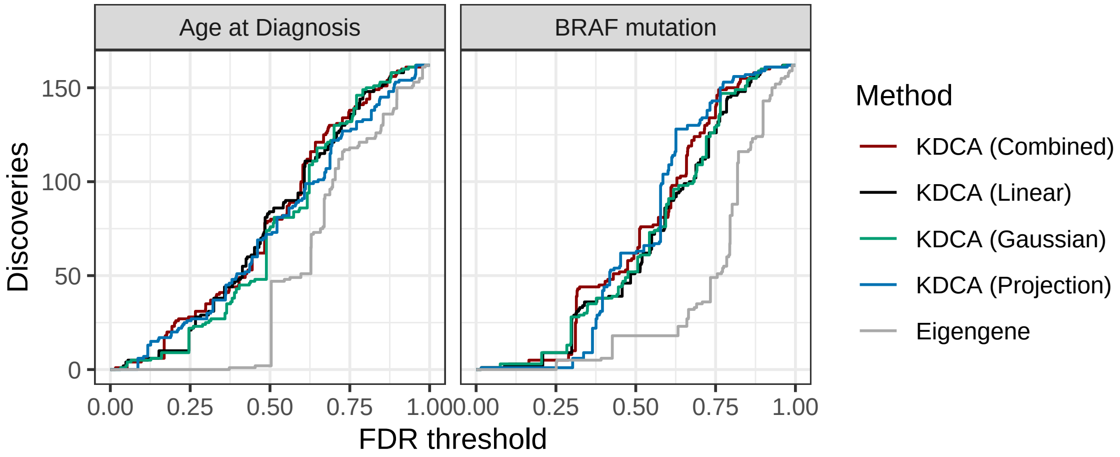

Figure S9: The number of discoveries as a function of FDR threshold using the Gaussian (green), linear (black), and projection (blue) kernels in the TCGA thyroid cancer data set using the BioCarta pathways. We implemented an aggregate version (red) to maximize power and compared KDCA to an eigengene approach (grey). We tested *BRAF* mutation status (right) and age of diagnosis (left) for differential co-expression.

3 Supplementary tables

Table S1: Significance results of the TCGA thyroid cancer data using age of diagnosis and *BRAF* mutation as the risk factors of interest. The p-values for KDCA (Combined) and an eigengene approach are reported for each pathway tested.

Pathway	Risk factor	Size	Tested size	KDCA (Combined)	Eigengene
IL2RB	*BRAF* mutation	38	20	0.0001	0.1351
PRION	Age at diagnosis	12	7	0.0001	0.0318
RAS	*BRAF* mutation	23	6	0.0008	0.0056
CDMAC	*BRAF* mutation	24	6	0.0011	0.7174
GLEEVEC	*BRAF* mutation	24	10	0.0013	0.0853
EDG1	Age at diagnosis	22	10	0.0018	0.8792
ECM	Age at diagnosis	20	12	0.0019	0.0419
CTCF	*BRAF* mutation	25	11	0.0033	0.0023
TFF	*BRAF* mutation	27	12	0.0036	0.0657
GSK3	Age at diagnosis	20	9	0.0048	0.0448
IL6	Age at diagnosis	22	10	0.0051	0.4815
KERATINOCYTE	*BRAF* mutation	60	16	0.0073	0.2704
SHH	*BRAF* mutation	18	6	0.0101	0.0506
TNFR1	*BRAF* mutation	36	8	0.0104	0.4462
INFLAM	Age at diagnosis	52	10	0.0115	0.2346
IGF1R	*BRAF* mutation	26	11	0.0118	0.0834
BARRESTIN	*BRAF* mutation	18	6	0.0119	0.4309
INTEGRIN	*BRAF* mutation	37	7	0.0134	0.8311
MITOCHONDRIA	Age at diagnosis	19	7	0.0135	0.0061
IL1R	Age at diagnosis	39	11	0.0143	0.2205
BLYMPHOCYTE	*BRAF* mutation	31	7	0.0145	0.0615
ETS	*BRAF* mutation	18	9	0.0156	0.9677
CDC25	*BRAF* mutation	10	5	0.0157	0.4619
IL6	*BRAF* mutation	22	10	0.0169	0.6753
SPPA	Age at diagnosis	17	8	0.0170	0.3774
RB	*BRAF* mutation	14	5	0.0177	0.4774
HER2	Age at diagnosis	24	9	0.0185	0.4008
TCR	*BRAF* mutation	46	17	0.0191	0.2490
IL7	*BRAF* mutation	16	10	0.0207	0.2317
CXCR4	*BRAF* mutation	21	6	0.0219	0.4296
MAPK	*BRAF* mutation	91	25	0.0262	0.1135
IGF1	*BRAF* mutation	22	11	0.0263	0.3047
ACH	*BRAF* mutation	15	5	0.0274	0.0985
ATM	Age at diagnosis	20	7	0.0279	0.5667
IL2	Age at diagnosis	23	10	0.0297	0.3477
TCR	Age at diagnosis	46	17	0.0305	0.7825
IL1R	*BRAF* mutation	39	11	0.0306	0.6060

TCYTOTOXIC	*BRAF* mutation	13	7	0.0310	0.3022
DC	*BRAF* mutation	17	8	0.0329	0.8813
IL7	Age at diagnosis	16	10	0.0341	0.6991
IL4	Age at diagnosis	11	5	0.0349	0.3675
AKT	*BRAF* mutation	22	7	0.0356	0.0948
DC	Age at diagnosis	17	8	0.0368	0.0078
RHO	Age at diagnosis	22	8	0.0411	0.0062
MTA3	Age at diagnosis	18	8	0.0414	0.9890
ARF	Age at diagnosis	17	7	0.0417	0.0372
ECM	*BRAF* mutation	20	12	0.0423	0.2781
KERATINOCYTE	Age at diagnosis	60	16	0.0426	0.4346
CXCR4	Age at diagnosis	21	6	0.0444	0.3752
TNFR2	Age at diagnosis	18	6	0.0455	0.1313
BARRESTIN	Age at diagnosis	18	6	0.0460	0.3105
KREB	Age at diagnosis	8	6	0.0466	0.0891
IL12	Age at diagnosis	19	7	0.0480	0.0017
P38MAPK	*BRAF* mutation	42	12	0.0483	0.0352
HER2	*BRAF* mutation	24	9	0.0494	0.0726
PPARA	*BRAF* mutation	64	21	0.0510	0.0829
TFF	Age at diagnosis	27	12	0.0584	0.3853
RACCYCD	*BRAF* mutation	27	9	0.0589	0.4235
CTL	Age at diagnosis	21	6	0.0594	0.0452
MAPK	Age at diagnosis	91	25	0.0597	0.5859
RACC	Age at diagnosis	16	7	0.0612	0.4771
NKT	*BRAF* mutation	31	11	0.0614	0.3763
EIF4	*BRAF* mutation	25	6	0.0635	0.1415
NGF	*BRAF* mutation	21	7	0.0645	0.0346
CTLA4	Age at diagnosis	38	12	0.0648	0.6556
IL2RB	Age at diagnosis	38	20	0.0648	0.1301
CDMAC	Age at diagnosis	24	6	0.0659	0.0408
CD40	Age at diagnosis	16	6	0.0676	0.0317
THELPER	Age at diagnosis	13	7	0.0687	0.2220
G2	Age at diagnosis	26	11	0.0700	0.3258
STRESS	Age at diagnosis	32	7	0.0713	0.5265
FCER1	*BRAF* mutation	41	15	0.0716	0.0586
CARDIACEGF	*BRAF* mutation	20	7	0.0717	0.2488
MAL	Age at diagnosis	20	8	0.0718	0.3188
INTEGRIN	Age at diagnosis	37	7	0.0756	0.1149
NFKB	Age at diagnosis	29	8	0.0756	0.6426
FAS	*BRAF* mutation	35	9	0.0763	0.4944
EGF	*BRAF* mutation	28	11	0.0797	0.0451
ERK5	Age at diagnosis	16	5	0.0807	0.0339
RECK	Age at diagnosis	10	5	0.0814	0.0145
MONOCYTE	Age at diagnosis	11	6	0.0820	0.1100
IL2	*BRAF* mutation	23	10	0.0822	0.3453

FCER1	Age at diagnosis	41	15	0.0844	0.0059
THELPER	*BRAF* mutation	13	7	0.0865	0.5205
PML	Age at diagnosis	30	5	0.0880	0.6394
CTL	*BRAF* mutation	21	6	0.0961	0.1401
AT1R	*BRAF* mutation	28	5	0.0998	0.1845
RACC	*BRAF* mutation	16	7	0.1012	0.2722
VITCB	Age at diagnosis	11	5	0.1069	0.6922
IL3	*BRAF* mutation	16	5	0.1115	0.1073
GHRELIN	*BRAF* mutation	13	7	0.1167	0.3847
HIVNEF	Age at diagnosis	69	15	0.1171	0.1939
EGF	Age at diagnosis	28	11	0.1172	0.5368
PTEN	*BRAF* mutation	20	5	0.1204	0.0292
G2	*BRAF* mutation	26	11	0.1211	0.7843
IL17	*BRAF* mutation	15	8	0.1220	0.0316
TOLL	Age at diagnosis	26	9	0.1222	0.1057
CERAMIDE	Age at diagnosis	21	8	0.1300	0.9140
EPHA4	Age at diagnosis	8	5	0.1329	0.6388
ERK5	*BRAF* mutation	16	5	0.1330	0.7123
TCRA	*BRAF* mutation	31	10	0.1330	0.1180
TCRA	Age at diagnosis	31	10	0.1353	0.1963
NFAT	*BRAF* mutation	55	15	0.1372	0.0810
TPO	*BRAF* mutation	25	8	0.1410	0.1136
PAR1	Age at diagnosis	19	8	0.1440	0.5047
INSULIN	*BRAF* mutation	23	12	0.1499	0.4030
AGR	Age at diagnosis	34	7	0.1530	0.4356
CD40	*BRAF* mutation	16	6	0.1581	0.1139
CTCF	Age at diagnosis	25	11	0.1588	0.5148
AMI	*BRAF* mutation	20	7	0.1610	0.2446
GPCR	*BRAF* mutation	33	9	0.1610	0.0609
NTHI	*BRAF* mutation	31	10	0.1640	0.0599
EDG1	*BRAF* mutation	22	10	0.1642	0.0820
MET	*BRAF* mutation	36	14	0.1646	0.0702
AMI	Age at diagnosis	20	7	0.1649	0.3220
CBL	Age at diagnosis	12	5	0.1659	0.7832
BAD	Age at diagnosis	27	16	0.1681	0.2220
P53HYPOXIA	Age at diagnosis	26	10	0.1699	0.1888
GH	*BRAF* mutation	30	12	0.1745	0.3275
EICOSANOID	*BRAF* mutation	23	14	0.1759	0.9639
GH	Age at diagnosis	30	12	0.1774	0.0787
TH1TH2	Age at diagnosis	40	8	0.1837	0.6705
RACCYCD	Age at diagnosis	27	9	0.1870	0.2568
BCR	*BRAF* mutation	35	9	0.1888	0.1288
TGFB	Age at diagnosis	19	7	0.1891	0.0858
GSK3	*BRAF* mutation	20	9	0.1912	0.1063
PCAF	*BRAF* mutation	14	6	0.1976	0.6697

CDC25	Age at diagnosis	10	5	0.1995	0.0364
BAD	*BRAF* mutation	27	16	0.2001	0.0751
MCALPAIN	*BRAF* mutation	21	9	0.2009	0.2583
IL5	Age at diagnosis	29	5	0.2027	0.4650
RB	Age at diagnosis	14	5	0.2031	0.0341
RECK	*BRAF* mutation	10	5	0.2038	0.1462
PDGF	*BRAF* mutation	29	12	0.2058	0.0751
DSP	Age at diagnosis	11	5	0.2125	0.1392
LONGEVITY	*BRAF* mutation	16	8	0.2129	0.6906
P38MAPK	Age at diagnosis	42	12	0.2131	0.7741
HDAC	*BRAF* mutation	26	7	0.2144	0.0820
HCMV	Age at diagnosis	17	5	0.2162	0.5390
IL10	Age at diagnosis	20	5	0.2217	0.5303
STRESS	*BRAF* mutation	32	7	0.2242	0.6999
CFTR	*BRAF* mutation	12	5	0.2248	0.0400
INTRINSIC	*BRAF* mutation	23	7	0.2260	0.2259
PAR1	*BRAF* mutation	19	8	0.2261	0.3640
D4GDI	*BRAF* mutation	12	8	0.2286	0.3203
NO1	Age at diagnosis	29	11	0.2297	0.4959
HIF	Age at diagnosis	16	5	0.2317	0.0898
ARF	*BRAF* mutation	17	7	0.2319	0.1615
PLCE	*BRAF* mutation	12	5	0.2328	0.0781
ACH	Age at diagnosis	15	5	0.2332	0.3970
ERYTH	Age at diagnosis	17	5	0.2348	0.6569
ALK	Age at diagnosis	38	13	0.2367	0.7388
AKAPCENTROSOME	Age at diagnosis	15	6	0.2370	0.1901
ALK	*BRAF* mutation	38	13	0.2397	0.1459
EXTRINSIC	Age at diagnosis	13	5	0.2416	0.2847
IL5	*BRAF* mutation	29	5	0.2479	0.1394
ERK	Age at diagnosis	29	7	0.2633	0.2046
PTDINS	*BRAF* mutation	23	5	0.2692	0.4074
CCR5	*BRAF* mutation	19	7	0.2726	0.4898
PDGF	Age at diagnosis	29	12	0.2783	0.8990
GRANULOCYTES	*BRAF* mutation	22	8	0.2833	0.3422
MHC	Age at diagnosis	66	8	0.2842	0.2632
MET	Age at diagnosis	36	14	0.2849	0.4504
MCM	Age at diagnosis	18	8	0.2854	0.0474
HIVNEF	*BRAF* mutation	69	15	0.2910	0.5785
NO2IL12	*BRAF* mutation	15	7	0.2934	0.9634
IL4	*BRAF* mutation	11	5	0.2945	0.4646
DEATH	*BRAF* mutation	30	9	0.2974	0.8963
BIOPEPTIDES	*BRAF* mutation	31	5	0.3037	0.5821
TOB1	Age at diagnosis	19	9	0.3041	0.9537
MHC	*BRAF* mutation	66	8	0.3085	0.3726
FMLP	Age at diagnosis	36	10	0.3121	0.7675

P53	Age at diagnosis	16	9	0.3122	0.5867
BIOPEPTIDES	Age at diagnosis	31	5	0.3201	0.3904
NTHI	Age at diagnosis	31	10	0.3206	0.2074
TOLL	*BRAF* mutation	26	9	0.3228	0.9623
ETS	Age at diagnosis	18	9	0.3315	0.1040
PLATELETAPP	*BRAF* mutation	14	6	0.3316	0.2267
VEGF	*BRAF* mutation	28	11	0.3347	0.3423
LAIR	*BRAF* mutation	24	9	0.3375	0.8209
RAC1	Age at diagnosis	21	13	0.3402	0.0850
G1	Age at diagnosis	28	12	0.3420	0.5244
CARDIACEGF	Age at diagnosis	20	7	0.3428	0.5546
CDK5	*BRAF* mutation	14	6	0.3513	0.3752
NPP1	*BRAF* mutation	10	5	0.3541	0.8971
BCR	Age at diagnosis	35	9	0.3547	0.0187
PTEN	Age at diagnosis	20	5	0.3547	0.2378
ARAP	*BRAF* mutation	17	6	0.3581	0.5179
NO1	*BRAF* mutation	29	11	0.3585	0.1879
NKT	Age at diagnosis	31	11	0.3595	0.9899
NUCLEARRS	Age at diagnosis	36	10	0.3603	0.5347
EXTRINSIC	*BRAF* mutation	13	5	0.3608	0.3660
TNFR1	Age at diagnosis	36	8	0.3616	0.9838
NGF	Age at diagnosis	21	7	0.3617	0.5883
MAL	*BRAF* mutation	20	8	0.3644	0.6809
HDAC	Age at diagnosis	26	7	0.3652	0.2814
G1	*BRAF* mutation	28	12	0.3685	0.5102
PPARA	Age at diagnosis	64	21	0.3709	0.4323
CHREBP	*BRAF* mutation	20	6	0.3829	0.0579
AT1R	Age at diagnosis	28	5	0.3841	0.2764
MPR	*BRAF* mutation	25	9	0.3842	0.2434
HCMV	*BRAF* mutation	17	5	0.3882	0.2140
PGC1A	Age at diagnosis	16	5	0.3908	0.3721
EFP	*BRAF* mutation	18	5	0.3933	0.7616
GPCR	Age at diagnosis	33	9	0.3945	0.4203
NPP1	Age at diagnosis	10	5	0.3959	0.7603
BCELLSURVIVAL	*BRAF* mutation	15	7	0.3969	0.8044
CASPASE	*BRAF* mutation	22	10	0.3974	0.6631
NKCELLS	*BRAF* mutation	29	8	0.4015	0.9193
SPRY	*BRAF* mutation	18	8	0.4019	0.1313
BBCELL	*BRAF* mutation	27	5	0.4057	0.1361
NO2IL12	Age at diagnosis	15	7	0.4069	0.1314
ASBCELL	*BRAF* mutation	31	5	0.4130	0.1362
CHREBP	Age at diagnosis	20	6	0.4221	0.2880
TGFB	*BRAF* mutation	19	7	0.4230	0.4151
VITCB	*BRAF* mutation	11	5	0.4258	0.6098
ARENRF2	Age at diagnosis	21	7	0.4285	0.1236

GCR	Age at diagnosis	17	5	0.4355	0.7621
FEEDER	Age at diagnosis	9	5	0.4363	0.5249
ERYTH	*BRAF* mutation	17	5	0.4415	0.2293
ASBCELL	Age at diagnosis	31	5	0.4435	0.5544
ARENRF2	*BRAF* mutation	21	7	0.4440	0.4342
BBCELL	Age at diagnosis	27	5	0.4445	0.5511
WNT	*BRAF* mutation	24	5	0.4455	0.2281
P53	*BRAF* mutation	16	9	0.4489	0.3859
NFAT	Age at diagnosis	55	15	0.4519	0.2907
LYM	Age at diagnosis	14	8	0.4529	0.8555
IGF1R	Age at diagnosis	26	11	0.4584	0.3561
CDK5	Age at diagnosis	14	6	0.4596	0.3971
PTDINS	Age at diagnosis	23	5	0.4637	0.2573
DREAM	Age at diagnosis	14	6	0.4709	0.6159
RAS	Age at diagnosis	23	6	0.4742	0.1869
VIP	*BRAF* mutation	27	8	0.4746	0.3606
CTLA4	*BRAF* mutation	38	12	0.4764	0.3528
INFLAM	*BRAF* mutation	52	10	0.4784	0.1421
HIF	*BRAF* mutation	16	5	0.4794	0.7887
FMLP	*BRAF* mutation	36	10	0.4797	0.2151
EFP	Age at diagnosis	18	5	0.4804	0.9628
AKT	Age at diagnosis	22	7	0.4827	0.8516
INTRINSIC	Age at diagnosis	23	7	0.4886	0.3953
STATHMIN	Age at diagnosis	21	9	0.5062	0.7917
CELLCYCLE	*BRAF* mutation	24	11	0.5065	0.6469
AHSP	Age at diagnosis	13	6	0.5076	0.6259
P53HYPOXIA	*BRAF* mutation	26	10	0.5079	0.7546
SPRY	Age at diagnosis	18	8	0.5083	0.7933
EPHA4	*BRAF* mutation	8	5	0.5137	0.6454
ERK	*BRAF* mutation	29	7	0.5152	0.2797
FAS	Age at diagnosis	35	9	0.5218	0.4286
PLATELETAPP	Age at diagnosis	14	6	0.5226	0.9100
CARM	*BRAF* mutation	26	6	0.5250	0.7879
IL12	*BRAF* mutation	19	7	0.5323	0.5173
ATM	*BRAF* mutation	20	7	0.5382	0.3500
CREB	*BRAF* mutation	25	9	0.5434	0.1365
DEATH	Age at diagnosis	30	9	0.5440	0.7224
EICOSANOID	Age at diagnosis	23	14	0.5454	0.1064
PITX2	*BRAF* mutation	16	5	0.5500	0.9688
MCALPAIN	Age at diagnosis	21	9	0.5645	0.4681
IGF1	Age at diagnosis	22	11	0.5648	0.5144
BLYMPHOCYTE	Age at diagnosis	31	7	0.5721	0.5845
CSK	*BRAF* mutation	40	13	0.5728	0.2521
LONGEVITY	Age at diagnosis	16	8	0.5743	0.5558
PITX2	Age at diagnosis	16	5	0.5849	0.1492

PGC1A	*BRAF* mutation	16	5	0.5927	0.4591
CELLCYCLE	Age at diagnosis	24	11	0.5947	0.8483
LYM	*BRAF* mutation	14	8	0.5977	0.5289
VIP	Age at diagnosis	27	8	0.5993	0.7770
TH1TH2	*BRAF* mutation	40	8	0.6011	0.3354
PCAF	Age at diagnosis	14	6	0.6037	0.5119
AHSP	*BRAF* mutation	13	6	0.6226	0.9261
IL3	Age at diagnosis	16	5	0.6256	0.4027
NUCLEARRS	*BRAF* mutation	36	10	0.6307	0.4813
PML	*BRAF* mutation	30	5	0.6311	0.7974
SHH	Age at diagnosis	18	6	0.6348	0.7601
WNT	Age at diagnosis	24	5	0.6370	0.7271
DREAM	*BRAF* mutation	14	6	0.6377	0.8255
NKCELLS	Age at diagnosis	29	8	0.6418	0.9475
D4GDI	Age at diagnosis	12	8	0.6458	0.1492
INSULIN	Age at diagnosis	23	12	0.6479	0.8250
RAC1	*BRAF* mutation	21	13	0.6538	0.3332
GHRELIN	Age at diagnosis	13	7	0.6539	0.7241
MITOCHONDRIA	*BRAF* mutation	19	7	0.6620	0.8246
VEGF	Age at diagnosis	28	11	0.6672	0.1995
PLCE	Age at diagnosis	12	5	0.6685	0.7181
PRION	*BRAF* mutation	12	7	0.6730	0.7931
IL17	Age at diagnosis	15	8	0.6768	0.2980
CSK	Age at diagnosis	40	13	0.6774	0.2093
CK1	Age at diagnosis	17	6	0.6794	0.3285
COMP	Age at diagnosis	43	7	0.6939	0.8751
AKAPCENTROSOME	*BRAF* mutation	15	6	0.6948	0.4653
CARM	Age at diagnosis	26	6	0.6969	0.8579
TCYTOTOXIC	Age at diagnosis	13	7	0.6986	0.1739
AGR	*BRAF* mutation	34	7	0.7111	0.2782
MONOCYTE	*BRAF* mutation	11	6	0.7130	0.2471
CREB	Age at diagnosis	25	9	0.7260	0.5402
KREB	*BRAF* mutation	8	6	0.7327	0.4926
GCR	*BRAF* mutation	17	5	0.7387	0.7088
NFKB	*BRAF* mutation	29	8	0.7394	0.4071
CBL	*BRAF* mutation	12	5	0.7422	0.3123
MCM	*BRAF* mutation	18	8	0.7578	0.2867
GRANULOCYTES	Age at diagnosis	22	8	0.7640	0.1574
CCR5	Age at diagnosis	19	7	0.7701	0.8300
EIF4	Age at diagnosis	25	6	0.7783	0.4263
CK1	*BRAF* mutation	17	6	0.7795	0.1702
BCELLSURVIVAL	Age at diagnosis	15	7	0.7868	0.7532
TNFR2	*BRAF* mutation	18	6	0.7918	0.2679
CASPASE	Age at diagnosis	22	10	0.7921	0.4017
CERAMIDE	*BRAF* mutation	21	8	0.8184	0.9860

MTA3	*BRAF* mutation	18	8	0.8232	0.5692
FEEDER	*BRAF* mutation	9	5	0.8238	0.0810
GLEEVEC	Age at diagnosis	24	10	0.8294	0.2467
TPO	Age at diagnosis	25	8	0.8320	0.8170
SPPA	*BRAF* mutation	17	8	0.8373	0.7480
CLASSIC	*BRAF* mutation	31	6	0.8429	0.4502
CFTR	Age at diagnosis	12	5	0.8480	0.7594
COMP	*BRAF* mutation	43	7	0.8663	0.6348
IL10	*BRAF* mutation	20	5	0.8747	0.9717
CLASSIC	Age at diagnosis	31	6	0.8765	0.3276
DSP	*BRAF* mutation	11	5	0.8820	0.5457
MPR	Age at diagnosis	25	9	0.8828	0.3696
RHO	*BRAF* mutation	22	8	0.9011	0.8182
LAIR	Age at diagnosis	24	9	0.9143	0.9318
TOB1	*BRAF* mutation	19	9	0.9199	0.7088
ARAP	Age at diagnosis	17	6	0.9696	0.5818
STATHMIN	*BRAF* mutation	21	9	0.9748	0.2347

References

1. Bass AJ, Bian S, Wingo AP, Wingo TS, Cutler DJ, Epstein MP. Identifying latent genetic interactions in genome-wide association studies using multiple traits. Genome Medicine. 2024;16(1):62.

2. Parsana P, Ruberman C, Jaffe AE, Schatz MC, Battle A, Leek JT. Addressing confounding artifacts in reconstruction of gene co-expression networks. Genome Biology. 2019;20(1):94.
